# New horizons in hypoxia signaling pathways

**DOI:** 10.1016/j.yexcr.2017.03.008

**Published:** 2017-07-15

**Authors:** Christopher W. Pugh, Peter J. Ratcliffe

**Affiliations:** a Target Discovery Institute, University of Oxford, OX3 7FZ, UK; b Francis Crick Institute, Midland Road, London NW1 1AT, UK

**Keywords:** Hypoxia, Protein hydroxylation, Prolyl hydroxylase, Hypoxia-inducible factor, Transcription, Cancer

## Abstract

Investigation into the regulation of the erythropoietin gene by oxygen led to the discovery of a process of direct oxygen sensing that transduces many cellular and systemic responses to hypoxia. The oxygen-sensitive signal is generated through the catalytic action of a series of 2-oxoglutarate-dependent oxygenases that regulate the transcription factor hypoxia-inducible factor (HIF) by the post-translational hydroxylation of specific amino acid residues. Here we review the implications of the unforeseen complexity of the HIF transcriptional cascade for the physiology and pathophysiology of hypoxia, and consider the origins of post-translational hydroxylation as a signaling process.

## 1 Introduction

Work over the last two to three decades has revealed a system of direct oxygen sensing that operates in essentially all animal cells, generating a new field of research into the biology of hypoxia, one to which Lorenz Poellinger made a range of important contributions. In this review we provide a brief background and, in the spirit of Lorenz's enigmatic enquiring mind, highlight some areas of ongoing intrigue.

Precise co-ordination of oxygen supply with demand is essential to meet the needs of metabolism and avoid toxicity. But despite intensive study of the physiology of oxygen delivery systems (the heart, lungs and blood circulation) for most of the twentieth century, the possibility of direct oxygen sensing was overlooked in favour of systems that respond to the products of energy metabolism. Remarkably it was the observation that exposure to cobalt induced erythrocytosis [1] that led to the concept of a specific oxygen sensor whose function was perturbed by cobaltous ions, but it was believed that this process was restricted to erythropoietin producing cells in liver and kidney. Initial clues that this system might operate more generally came from the observation that very small quantities of erythropoietin mRNA were expressed outside the liver and kidneys, but also induced by physiological hypoxia [2]. However, the first clear evidence that the same molecular pathways underlying erythropoietin regulation operated widely across mammalian cells came from transfection studies which revealed that oxygen-regulated control sequences derived from the erythropoietin gene operated widely in non-erythropoietin producing cells [3]. This work was closely followed by demonstration of the general operation of the key transcription factor hypoxiainducible factor (HIF) [4] and by the identification of genes encoding glycolytic enzymes as the first genes other than erythropoietin regulated through this pathway [5], [6]. Rapidly, it became clear that the repertoire of HIF target genes was very large. Successive advances in genetic and genomic analysis revealed yet greater complexity and in complete contrast to the entry point through erythropoietin, hundreds of genes are now known to respond directly or indirectly to hypoxia via HIF.

HIF binds to DNA as a heterodimer of one alpha isoform with one beta isoform, each of which is a basic-helix-loop-helix PAS (Per-Ahr/ARNT-Sim) protein [7]. Elucidation of the mechanism of oxygen sensing revealed a remarkably direct process which again was entirely unanticipated. In the presence of oxygen, hydroxylation of two specific prolyl residues in HIF-alpha chains promotes interaction with the von Hippel-Lindau ubiquitin (VHL) E3 ligase leading to ubiquitylation and hence proteasomal destruction [8], [9], [10]. In human cells, three closely related enzymes PHD (prolyl hydroxylase domain) 1, 2 and 3 catalyse this reaction [11], [12]. In a second oxygen-sensitive system asparaginyl hydroxylation at a site within the C-terminal activation domain of HIF-alpha [13], catalyzed by a more distantly related enzyme FIH (factor inhibiting HIF), impairs the recruitment of co-activators and downregulates HIF transcriptional activity (reviewed in [14]). These enzymes are all 2-oxoglutarate-dependent oxygenases that split dioxygen and incorporate one atom of oxygen into the HIF substrate, coupling this reaction to the oxidative decarboxylation of 2-oxoglutarate, yielding succinate and carbon dioxide. In hypoxia, these reactions are suppressed leading HIF-alpha to escape destruction and assemble into an active transcriptional complex. The hydroxylation reaction is also powerfully inhibited by cobaltous ions, accounting for the founding toxicological observations [11].

## 2 More than oxygen homeostasis or a new physiology of hypoxia?

**Fig. 1:**
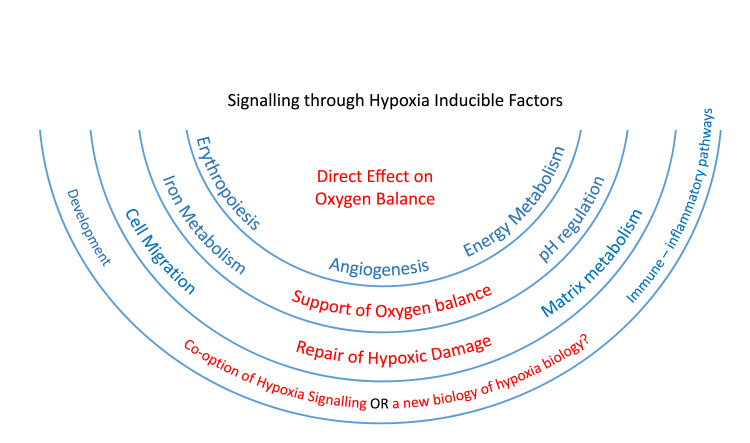
Schematic illustrating the operation of the HIF pathway on processes both directly and indirectly related to the maintenance of oxygen balance. The HIF targets first identified were genes with direct effects on oxygen balance but as the number of identified HIF targets has increased the links of many to oxygen homeostasis have become increasingly indirect as exemplified by those involved in development and immune-inflammatory pathways.

Many of the earliest HIF targets genes to be identified, encoding erythropoietin, angiogenic growth factors, and enzymes catalysing pathways of energy metabolism, had clear functions in maintaining oxygen balance. Others, such as those encoding molecules involved in iron transport or pH regulation had functions that could be connected indirectly with oxygen metabolism, and HIF became known as the master regulator of oxygen homeostasis. Interestingly, as the number of identified targets of HIF has expanded the links of many to oxygen homeostasis have become increasingly indirect (Fig. 1).

An important inflection point was the discovery, using genetic inactivation of HIF-1alpha in myeloid cells, of effects in inflammatory systems that extend well beyond the conventional boundaries of oxygen physiology [15]. Most recently pan-genomic studies have identified hundreds of direct HIF transcriptional targets whose products operate in an extraordinarily wide range of cellular processes [16]. Together with the operation of HIF on microRNA [17] and long non-coding RNA networks [18] and on multiple epigenetic and secondary transcriptional cascades [14], these studies suggest that the pathway impinges on almost every area of cell biology and extends into numerous areas that were previously unconnected with the physiology of hypoxia. In parallel it has become clear that HIF can be induced by a range of non-hypoxic stimuli, including growth, differentiation, metabolic, inflammatory and immune signals.

These unanticipated findings raise new questions. Do they represent a new physiology of hypoxia, in which a wide range of processes are dynamically controlled by oxygen availability, do they reflect co-option of non-hypoxia HIF signaling for different purposes, or do they reflect a permissive dependence on the integrity of the pathway? These possibilities are of course not mutually exclusive. Intriguingly, many of the newly defined processes are anatomically localised to areas of physiological hypoxia or occur in zones of pathological hypoxia. For instance, stem cell populations often localise to hypoxic micro-environments (reviewed in [19]). Marked zonal hypoxia is observed within the thymus and lymphoid tissues [20]. Dense cellular infiltrates and impaired vascular delivery create profound hypoxia in inflamed tissues (reviewed in [21]). It is likely that micro-environmental hypoxia itself contributes to the activation of HIF in many of these settings. However, an important, and largely unanswered question concerns the extent to which these processes are affected by systemic hypoxia, such as occurs in diseases that affect oxygen delivery, or at altitude. Attempts to date to address this question have yielded conflicting answers. On the one hand, studies using direct measurement of tissue pO2 have suggested that many local zones of physiological hypoxia are little influenced by systemic hypoxia [22]. Other studies have measured HIF directly and revealed biologically important effects [23]. For instance, systemic hypoxia clearly induces HIF in the renal papilla where the normal ‘physiological’ pO2 is already so low that a further reduction must necessarily be small. This suggests, at least in this setting, that very small changes in local pO2, which may be difficult to measure physically, have the potential to exert biological effects through the HIF pathway [23].

Given the role of HIF signaling in so many processes, this question of interplay between systemic and local hypoxia is relevant to many aspects of physiology and medicine. Does developmental hypoxia constrain development itself, if so does systemic hypoxia impinge on development? Does sojourn at altitude affect the function of immune/inflammatory pathways in ways that could affect disease susceptibility or (as was once popular) recovery from disease? The relevance of these questions is further underscored by the development of therapy that aims to modulate these pathways. The most advanced clinical application is that of HIF prolyl hydroxylase inhibitors that aim to activate HIF in the treatment of anaemia associated with deficient erythropoietin production in kidney disease (reviewed in [24]). The pharmacokinetic concentration of drugs in the liver and kidneys, together with the intrinsically high sensitivity of the erythropoietin gene to this system, might permit relatively selective action on erythropoiesis. Nevertheless, it is likely that clinical exposure to such agents in the setting of renal anaemia will impinge, for better or worse, on inflammatory disease, at least within the kidneys. Thus, a better understanding of whether and in what way small dose-dependent systemic activation of HIF has the potential to modulate the many new targets of the HIF system, is urgently required.

## 3 Un-physiological ‘switching’ of the HIF pathway in cancer

The massive interconnectivity of HIF pathways also has important implications in pathology. Much has been written about the constraints of highly connected molecular systems on species evolution. At the level of the somatic cell massive pathway connectivity has related implications which have received much less attention.

In general, it would be expected that responses to common pathologies experienced early in life would be subject to similar evolutionary pressures as other aspects of integrated physiology and that they could be viewed as physiological from the perspective of evolution. However, this might not be true of pathologies such as cancer that are more common after reproductive life. Tissue hypoxia is a common feature of cancer and HIF is frequently upregulated in cancer both by micro-environmental hypoxia and by links to oncogenic growth pathways. The latter most likely reflect physiological connections between the oxygen demands of growth and the role of HIF in maintaining oxygen homeostasis. As such, activation of HIF in cancer may often be quasi-physiological, supporting tumour growth, just as it supports physiological oxygen homeostasis.

However, the activation of growth pathways in cancer also occurs though stochastic effects of mutation, which may activate connected pathways in very un-physiological ways. A striking example is the activation of HIF by inactivating mutations of the VHL tumour suppressor, which is observed in renal cell carcinoma (RCC), which leads to high level activation of HIF irrespective of other considerations [8]. In this circumstance, the pathway is clearly operating outside the evolutionary constraints that shaped it. It is also unlikely the survival interests of the cell would map well onto those of the organism. Therefore, at the cellular level, massive functional connectivity may be predicted to generate adverse (anti-tumourigenic) as well as supportive (pro-tumourigenic) effects. Under such a model it would be the balance of these that determines oncogenic drive. This balance may be highly context specific, as is suggested by the organ specificity of VHL-associated cancer. Furthermore, there is no *a priori* reason for individual pro- and anti-tumourigenic events to be quantitatively similar. Thus it is unclear whether the interface of HIF with cancer is oligo-genic or polygenic, nor whether individual effects are quantitatively large or small. Given that pan-genomic studies have revealed that many other pathways that are activated in cancer are highly interconnected, such considerations would allow that many or even most phenotypes which are associated with cancer are not actually driving it, but simply co-selected by pathway interconnectivity. It could be argued that such a position is supported by the mismatch between plausible cell biology and genetic evidence for causality in many cancer phenotypes.

In discussion of these issues, Lorenz, in his inimitable way, pointed out that in the absence of direct evidence, these arguments are essentially philosophical. So what is the evidence? At least in the setting of VHL-associated cancer, we consider that there is persuasive evidence for pro- and anti-tumourigenic actions of different components of the HIF pathway. A substantial body of work suggests that in this setting the two major HIF-alpha isoforms HIF-1 and HIF-2 have respectively anti- and pro-tumourigenic effects [25], [26], [27]. Furthermore, within the target repertoire of each HIF isoform, there appear to be multiple pro- and anti-tumourigenic effects, as evidenced by functional annotation, and by multiple positive and negative contributions of the expression of transcriptional targets of HIF-1 or HIF-2 to the prediction of VHL-associated cancer outcome [28]. A central implication of this model is that the HIF pathway will be under selection to create a more oncogenic profile and to extinguish anti-tumourigenic components during cancer development. In RCC this is supported by the evolution from dominantly HIF-1 expression in the renal tubular epithelium to dominantly HIF-2 expression in RCC [29], by the existence of inactivating somatic mutations in HIF-1alpha in RCC [26], [30] and by the alignment of human RCC-susceptibility GWAS polymorphisms, with multiple components of the HIF transcriptional pathway [31], [32].

These findings strongly suggest that un-physiological activation of large interconnected pathways in cancer, as exemplified by HIF, creates selective pressures akin to those that operate on interconnected systems during species evolution. A key difference is that, in cancer cells, such selective pressures likely have the potential to drive escape mechanisms that enable tolerance of the adverse effects of co-selected pathways [33]. Such mechanisms might then generate the flexibility and heterogeneity that enables many tumours to avoid chemotherapeutic eradication. Though the un-physiological ‘switch’ of the HIF pathway following VHL-inactivation provides a good paradigm to study these effects, we argue that VHL inactivation is unlikely to be the only un-physiological switch affecting HIF in cancer, and unlikely that the HIF system is the only highly interconnected pathway that is ‘switched’ un-physiologically in cancer.

**Fig. 2:**
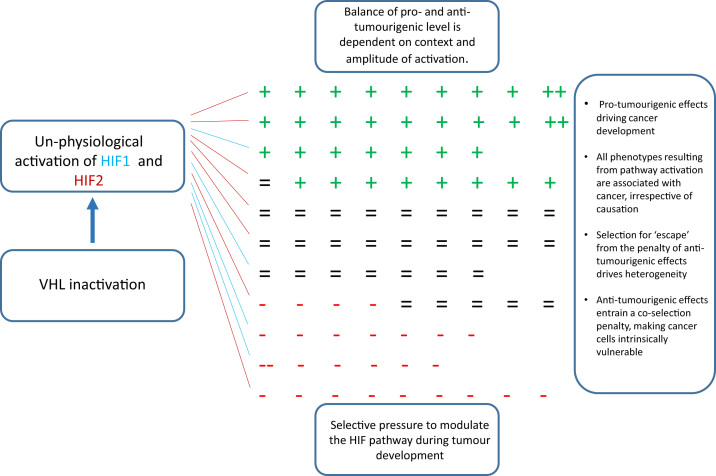
The implications of un-physiological ‘switching’ of massively interconnected pathways in cancer, exemplified by the activation of HIF following inactivation of the von Hippel-Lindau tumour suppressor. Inactivation of the von Hippel Lindau tumour suppressor leads to the unphysiological activation of both HIF-1 and HIF-2, each of which regulates its own set of target genes. Individual target genes will have net pro- (+), anti- (-) or broadly neutral (=) effects on tumour cell growth of varying intensity (+ or ++; - or --). In some contexts (i.e. those tissues where tumours do not arise in VHL disease) the net effect of this genetic switch will not be compatible with cell survival. In those tissues where tumours do arise in VHL disease the initial genetic switch is permissive for cell survival but many of the up-regulated genes will be co-selected passengers, rather than drivers of the cancer and the co-selected anti-tumourigenic genes will make the cells intrinsically vulnerable. It is proposed that the genetic switch will alter the cellular milieu and further selective pressures will lead to the enhanced representation of cells with altered gene expression profiles that qualitatively or quantitatively favour pro-tumourigenic genes and /or down-regulate anti-tumourigenic genes. This selection may act at the level of individual genes or entire sub-pathways, as exemplified by the tendency to silence or inactivate HIF-1 alpha in renal cancer. At any one time a tumour will contain a heterogeneous mixture of cells exhibiting different solutions compatible with survival.

In our view these considerations merit a rebalance of thinking in cancer research towards consideration of massive pathway connectivity, the consequent contextual specificity of selective mechanisms, and the events in cancer development that might be predicted by this model to license tumour evolution by enabling escape from the ‘co-selection’ penalty (Fig. 2).

## 4 Perplexing patterns of protein oxidation in signaling hypoxia

The ‘HIF hydroxylase’ oxygen signaling pathway appears to be a universal feature of animal life. Genomic analyses have revealed at least one isoform of each component of the HIF-PHD-VHL triad in every animal species [34]. Most primitive invertebrates possess a single set of proteins, with multiple isoforms arising through gene duplication events at the base of vertebrate evolution. The primitive HIF-PHD couple most closely resembles the most abundant proteins in mammalian cells, PHD2 and HIF-1alpha, other isoforms being tissue restricted or expressed at lower levels. Oddly (given the general importance of oxygen to life) the integrated HIF-PHD-VHL system has so far only been defined in animal species. However, each individual component and the principal mode of operation (oxygen-dependent protein degradation) is observed much more widely across different kingdoms. Moreover, distinct domains in HIF-alpha polypeptides can be traced to more ancient environmental sensing pathways.

HIF is a basic-helix-loop-helix PAS protein. Basic-helix-loop-helix (bHLH) transcription factors are widely represented across eukaryotic kingdoms. The PAS domain can be identified even more widely across both prokaryotes and eukaryotes, but bHLH-PAS transcription factors are apparently restricted to animals. PAS domains have been shown to transduce a wide range of environmental signals including oxygen levels [35]. In HIF, the function of the PAS domain (beyond its structural role in dimerization) is still unclear. Using fusion proteins it has been clearly demonstrated that PHD catalyzed hydroxylation of sites lying C-terminal to the PAS domain is necessary and sufficient for regulation by oxygen. However, in the related bHLH-PAS transcription factor the aryl hydrocarbon receptor, which co-ordinates the xenobiotic transcriptional response through dimerization with HIF-1beta [36], the PAS domain is the ligand binding domain. HIF-alpha proteins, particular HIF-2alpha, contain a conserved pocket in the PAS B domain suggestive of a ligand binding function, which has been used to design dimerization inhibitors [37]. Currently, it is unclear whether the PAS domains in HIF proteins have some other as yet undefined function through endogenous ligand binding, or have simply evolved to a structural role in this setting.

Though prolyl hydroxylation was well established as a structural modification of procollagen, its use in signaling as in the HIF hydroxylase system was at the time of its discovery unprecedented. However non-animal species possess similar enzymes, at least some of which have recently been characterized as signaling enzymes. Since the discovery of the HIF prolyl hydroxylases, two related prolyl hydroxylases have been well characterized as oxygen sensors. Thus, in *Dictyostelium discoideum*, a PHD-like enzyme catalyzes the hydroxylation of a prolyl residue on Skp1. The hydroxyproline residue then becomes the substrate for glycosylation. These modifications are proposed to promote association with other components of a Skp-Cullin-F box, E3 ubiquitin ligase, which results in the ubiquitylation and degradation of proteins that control *Dictyostelium* development [38]. In animal cells, Skp1 orthologues also function as components of SCF ubiquitin E3 ligases. However, to date, SCF complexes have not been identified as hydroxylation targets and in mammalian species the target prolyl residue in Skp1 is not conserved. Nevertheless, the *Dictyostelium* oxygen sensing system has intriguing similarities with the animal PHD-HIF-VHL pathway, in which VHL also complexes (with elongins B and C) in an SCF-like ubiquitin ligase, but regulatory hydroxylation occurs on the substrate (HIF) as opposed to the ligase.

Also intriguing are the cross-species relationships of another oxygen sensing prolyl hydroxylase, variously named Ofd1, *S*chizosaccharomyces pombe; Tpa1p, *Saccharomyces cerevisiae*; Sudestada, *Drosophila melanogaster*; OGFOD1, *Homo sapiens*. This enzyme is the most closely related human enzyme to the PHDs. In *S. pombe* Ofd1 functions as an oxygen sensor, its catalytic activity being essential for the oxygen-dependent degradation of the N-terminal portion of the sterol responsive transcription factor, Sre1N. Ofd1 itself consists of an N-terminal catalytic (2-oxoglutarate oxygenase) domain and a C-terminal domain that is necessary for proteolysis of Sre1N [39]. The hydroxylation substrate in this process is not clear; Sre1N has not itself been identified as a substrate and its degradation is presumably controlled indirectly by the hydroxylation of another protein. Despite strong conservation of Ofd1 as OGFOD1 in mammalian cells, we have not to date observed oxygen-regulation of the orthologous SREBPs. Rather these pathways appear to be indirectly connected to HIF. For instance, it has been reported that the basic-helix-loop-helix protein bHLHE40, itself a transcriptional target of HIF, acts as a transcriptional repressor of SREBP-1c at the promoter of the fatty acid synthase gene [40]. Thus, despite conservation of both the catalytic domain and the degradation domain of Ofd1, and conservation of the oxygen-regulated expression of some of its downstream effectors, the linking pathway does not appear to be conserved. Remarkably, however, one catalytic function is highly conserved. OGFOD1 and its orthologues catalyse trans-3-hydroxylation of a conserved prolyl residue in the small ribosomal sub-unit protein RPS23, which lies at the coding centre of the assembled ribosome [41], [42]. In keeping with this, inactivation results in context specific decoding effects. At present it is unclear whether this process has a physiological role in regulating responses to hypoxia, nor how it impinges on regulation of SreN in *S. pombe*. Interestingly however, despite being a ribosomal hydroxylase, OGFOD1 is a nuclear protein. One appealing but unproven hypothesis is that it functions in some way to co-ordinate ribosome biogenesis with a transcriptional function in biosynthesis.

Despite the uncertainties, these studies have revealed that at least two non-animal oxygen sensing systems use prolyl hydroxylation to signal hypoxia and both also transduce the signal via oxygen-regulated proteolysis. Yet another intriguing parallel is with oxygen sensing in plants. Plants make a wide variety of metabolic and morphological responses to hypoxia, which is often experienced in the context of flooding. So far, no HIF-like system has been defined. Rather the major transcriptional mediators of responses to hypoxia are Group-VII ethylene response (ERF-VII) transcription factors. Interestingly, as with HIF, these transcription factors are controlled by oxygen-regulated proteolysis. Following removal of methionine from the N-terminus of ERF-VII transcription factors, cysteine in position 2 is oxidized to cysteine sulfonic acid by the plant cysteine oxidases [43]. This makes the polypeptide a substrate for arginyl transferases that add an N-terminal arginine residue, which is a destabilizing residue in the N-terminal degradation pathway. In animals, orthologous oxygen sensing cysteine oxidases have not so far been defined, but a similar process of oxygen-sensitive cysteine oxidation has been proposed to occur non-enzymatically and to confer sensitivity to both oxygen and nitric oxide levels on a class of protein substrates containing MC followed by a basic residue at the N-terminus [44]. The evolutionary relationships amongst these processes are not clear. However, at least in the case of the plant system, which uses a different type of ‘oxygen sensing’ dioxygenase and ubiquitin ligase from the animal system, convergent evolution appears likely. Convergent evolution, in which similar solutions to the common problem of oxygen sensing have evolved independently, most likely also accounts for the two different types of oxygen-sensitive protein hydroxylation on HIF itself. As opposed to the PHDs, the HIF asparaginyl hydroxylase, FIH, belongs to a structurally different group of 2-oxoglutarate oxygenases. It catalyzes oxygen-sensitive hydroxylation in the C-terminus of human HIF-1alpha and HIF-2alpha, which regulates the transcriptional activity of HIF and has been proposed to ‘tune’ the oxygen sensitivity of the HIF transcriptional response. Notably FIH remains active at lower dioxygen levels than the HIF prolyl hydroxylases [45]. In theory, this would enable it to modulate the activity of HIF that escapes hydroxylation when the activity of the PHDs is suppressed, essentially extending the range of oxygen sensitivity.

However, FIH appears to be a more ‘promiscuous’ enzyme than the PHDs. In addition to HIF it hydroxylates a wide range of ankyrin repeat domain (ARD) containing proteins, targeting asparaginyl residues that are part of the ankyrin consensus [46]. In some ARD-containing proteins it can also hydroxylate other residues, including aspartatyl and histidinyl, when they appear in equivalent positions in the ankyrin repeat. The physiological role(s) of FIH-dependent hydroxylation of ARD-containing proteins are not yet clear. Unlike the PHDs, inactivation of FIH in the mouse is not associated with a phenotype suggestive of HIF activation, supporting its involvement in distinct biological circuitry [47].

**Fig. 3:**
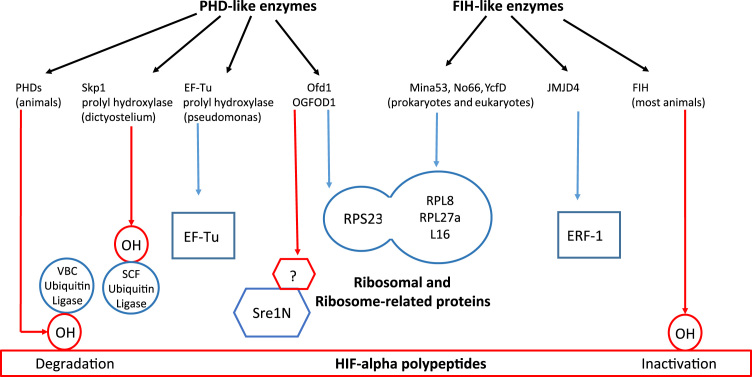
Multiple connections between oxygen sensors signaling to nuclear transcriptional functions and similar enzymes catalysing the hydroxylation of ribosomal and ribosome-related proteins. Intriguingly the sub-families of 2-oxoglutarate-dependent oxygenases that are most closely linked to the HIF prolyl (PHD) and asparginyl hydroxylases (FIH) both contain members that also target elongation factors (EF-Tu), release factors (ERF-1) and ribosomal proteins in addition to transcription factors. Known physiological oxygen-sensitive hydroxylations are indicated in red.

As with the PHD enzymes, the human genome encodes a number of enzymes that are similar to FIH and are predicted to catalyse protein hydroxylation. Remarkably, biochemical studies of several of these enzymes (Mina 53, No66, JmJd4) have recently been shown to catalyse hydroxylation of ribosomal (RPL27, RPL8) [48] and ribosome-related (ERF1) proteins [49]. These functions are again very highly conserved, with the prokaryotic enzyme, ycfD (structurally related to Mina53 and No66) catalysing arginyl hydroxylation at a related site in the prokaryotic ribosome [48], [50]. These findings raise yet another conundrum; that closely related members of each of two groups of distantly related 2-oxoglutarate oxygenases each catalyse either ribosomal hydroxylation, or HIF hydroxylation, appears unlikely to be a chance occurrence (Fig. 3). Does the co-incidence derive from some ancient link between oxygen sensing and the regulation of protein synthesis? Though the function is not yet clear, some prokaryotes contain PHD-like enzymes, the *Pseudomonas* enzyme PPHD being able to catalyse prolyl hydroxylation of elongation factor EF-Tu [51].

Neither the evolutionary nor the functional relationships amongst these processes are easy to tease out. Nevertheless the existence of these puzzling connections; PAS domains juxtaposed to oxygen-sensitive degradation domains, hydroxylation at key positions in the ribosome catalyzed by enzymes that resemble the HIF hydroxylases, common use of different (and energetically expensive) systems of protein oxidation and proteolysis to control gene expression, is clear. Whether these findings hold clues to new types of signaling pathway, or whether they simply represent evolutionary history is not clear. It is the job of biologists to navigate these tortuous pathways, something that requires an enquiring mind with a keen appetite for the unexpected. Sadly, in the passing of Lorenz Poellinger, the field has lost such an individual.

## References

[1] Waltner K., Waltner K. (1929). Kobalt und Blut. Klin. Wochenschr..

[2] Tan C.C., Eckardt K.-U., Firth J.D., Ratcliffe P.J. (1992). Feedback modulation of renal and hepatic erythropoietin mRNA in response to graded anemia and hypoxia. Am. J. Physiol..

[3] Maxwell P.H., Pugh C.W., Ratcliffe P.J. (1993). Inducible operation of the erythropoietin 3′ enhancer in multiple cell lines: evidence for a widespread oxygen sensing mechanism. Proc. Natl. Acad. Sci. USA.

[4] Wang G.L., Semenza G.L. (1993). General involvement of hypoxia-inducible factor 1 in transcriptional response to hypoxia. Proc. Natl. Acad. Sci. USA.

[5] Firth J.D., Ebert B.L., Pugh C.W., Ratcliffe P.J. (1994). Oxygen-regulated control elements in the phosphoglycerate kinase 1 and lactate dehydrogenase A genes: similarities with the erythropoeitin 3′ enhancer. Proc. Natl. Acad. Sci. USA.

[6] Semenza G.L., Roth P.H., Fang H.-M., Wang G.L. (1994). Transcriptional regulation of genes encoding glycolytic enzymes by hypoxia-inducible factor 1. J. Biol. Chem..

[7] Wang G.L., Jiang B.-H., Rue E.A., Semenza G.L. (1995). Hypoxia-inducible factor 1 is a basic-helix-loop-helix-PAS heterodimer regulated by cellular O_2_ tension. Proc. Natl. Acad. Sci. USA.

[8] Maxwell P.H. (1999). The tumour suppressor protein VHL targets hypoxia-inducible factors for oxygen-dependent proteolysis. Nature.

[9] Jaakkola P. (2001). Targeting of HIF-a to the von Hippel-Lindau ubiquitylation complex by O_2_-regulated prolyl hydroxylation. Science.

[10] Ivan M. (2001). HIFa targeted for VHL-mediated destruction by proline hydroxylation: implications for O2 sensing. Science.

[11] Epstein A.C.R. (2001). *C. elegans* EGL-9 and mammalian homologues define a family of dioxygenases that regulate HIF by prolyl hydroxylation. Cell.

[12] Bruick R.K., McKnight S.L. (2001). A conserved family of prolyl-4-hydroxylases that modify HIF. Science.

[13] Lando D., Peet D.J., Whelan D.A., Gorman J.J., Whitelaw M.L. (2002). Asparagine hydroxylation of the HIF transactivation domain: a hypoxic switch. Science.

[14] Lendahl U., Lee K.L., Yang H., Poellinger L. (2009). Generating specificity and diversity in the transcriptional response to hypoxia. Nat. Rev. Genet..

[15] Cramer T. (2003). HIF-1a is essential for myeloid cell-mediated inflammation. Cell.

[16] Schödel J. (2012). High-resolution genome-wide mapping of HIF-binding sites by ChIP-seq. Blood.

[17] Kulshreshtha R. (2007). A microRNA signature of hypoxia. Mol. Cell. Biol..

[18] Choudhry H. (2014). Extensive regulation of the non-coding transcriptome by hypoxia: role of HIF in releasing paused RNApol2. EMBO Rep..

[19] Simon M.C., Keith B. (2008). The role of oxygen availability in embryonic development and stem cell function. Nat. Rev. Mol. Cell Biol..

[20] Hale L.P., Braun R.D., Gwinn W.M., Greer P.K., Dewhirst M.W. (2002). Hypoxia in the thymus: role of oxygen tension in thymocyte survival. Am. J. Physiol. Heart Circ. Physiol..

[21] Eltzschig H.K., Carmeliet P. (2011). Hypoxia and inflammation. New Engl. J. Med..

[22] Schaffer L., Vogel J., Breymann C., Gassmann M., Marti H.H. (2006). Preserved placental oxygenation and development during severe systemic hypoxia. Am. J. Physiol. Regul. Integr. Comp. Physiol..

[23] Rosenberger C. (2002). Expression of hypoxia-inducible factor-1a and -2a in hypoxic and ischemic rat kidneys. J. Am. Soc. Nephrol..

[24] M.C. Chan, J.P. Holt-Martyn, C.J. Schofield, P.J. Ratcliffe, Pharmacological targeting of the HIF hydroxylases - a new field in medicine development. Mol. Aspects Med., vol. 47-48, 2016, pp. 54–75. 10.1016/j.mam.2016.01.001 26791432

[25] Raval R.R. (2005). Contrasting Properties of Hypoxia-Inducible Factor 1 (HIF-1) and HIF-2 in von Hippel-Lindau-Associated Renal Cell Carcinoma. Mol. Cell. Biol..

[26] Shen C. (2011). Genetic and functional studies implicate HIF1alpha as a 14q kidney cancer suppressor gene. Cancer Discov..

[27] Keith B., Johnson R.S., Simon M.C. (2012). HIF1alpha and HIF2alpha: sibling rivalry in hypoxic tumour growth and progression. Nat. Rev. Cancer.

[28] Salama R. (2015). Heterogeneous effects of direct hypoxia pathway activation in kidney cancer. PLoS One.

[29] Mandriota S.J. (2002). HIF activation identifies early lesions in VHL kidneys: evidence for site-specific tumor suppressor funtion in the nephron. Cancer Cell.

[30] Morris M.R. (2009). Mutation analysis of hypoxia-inducible factors HIF1A and HIF2A in renal cell carcinoma. Anticancer Res..

[31] J. Schödel, et al., Common genetic variants at the 11q13.3 renal cancer susceptibility locus influence binding of HIF to an enhancer of cyclin D1 expression. Nature Genet., vol. 44, 2012, pp. 420–425. 10.1038/ng.2204 PMC3378637 22406644

[32] Grampp S. (2016). Genetic variation at the 8q24.21 renal cancer susceptibility locus affects HIF binding to a MYC enhancer. Nat. Commun..

[33] Graeber T.G. (1994). Hypoxia induces accumulation of p53 protein, but activation of a G_1_-phase checkpoint by low-oxygen conditions is independent of p53 status. Mol. Cell. Biol..

[34] Loenarz C. (2011). The hypoxia-inducible transcription factor pathway regulates oxygen sensing in the simplest animal, Trichoplax adhaerens. EMBO Rep..

[35] Gu Y.-Z., Hogenesch J.B., Bradfield C.A. (2000). The PAS superfamily: sensors of environmental and developmental signals. Annu. Rev. Pharmacol. Toxicol..

[36] Gradin K. (1996). Functional interference between hypoxia and dioxin signal transduction pathways: competition for recruitment of the Arnt transcription factor. Mol. Cell. Biol..

[37] Scheuermann T.H. (2013). Allosteric inhibition of hypoxia inducible factor-2 with small molecules. Nat. Chem. Biol..

[38] West C.M., Blader I.J. (2015). Oxygen sensing by protozoans: how they catch their breath. Curr. Opin. Microbiol..

[39] Hughes B.T., Espenshade P.J. (2008). Oxygen-regulated degradation of fission yeast SREBP by Ofd1, a prolyl hydroxylase family member. EMBO J..

[40] Choi S.M. (2008). Stra13/DEC1 and DEC2 inhibit sterol regulatory element binding protein-1c in a hypoxia-inducible factor-dependent mechanism. Nucleic Acids Res..

[41] Singleton R.S. (2014). OGFOD1 catalyzes prolyl hydroxylation of RPS23 and is involved in translation control and stress granule formation. Proc. Natl. Acad. Sci. USA.

[42] Loenarz C. (2014). Hydroxylation of the eukaryotic ribosomal decoding center affects translational accuracy. Proc. Natl. Acad. Sci. USA.

[43] Weits D.A. (2014). Plant cysteine oxidases control the oxygen-dependent branch of the N-end-rule pathway. Nat. Commun..

[44] Hu R.G. (2005). The N-end rule pathway as a nitric oxide sensor controlling the levels of multiple regulators. Nature.

[45] Tian Y. (2011). Differential sensitivity of HIF hydroxylation sites to hypoxia and hydroxylase inhibitors. J. Biol. Chem..

[46] Cockman M.E. (2006). Posttranslational hydroxylation of ankyrin repeats in IkappaB proteins by the hypoxia-inducible factor (HIF) asparaginyl hydroxylase, factor inhibiting HIF (FIH). Proc. Natl. Acad. Sci. USA.

[47] Zhang N. (2010). The asparaginyl hydroxylase factor inhibiting HIF-1alpha is an essential regulator of metabolism. Cell Metab..

[48] Ge W. (2012). Oxygenase-catalyzed ribosome hydroxylation occurs in prokaryotes and humans. Nat. Chem. Biol..

[49] T. Feng, et al., Optimal Translational Termination Requires C4 Lysyl Hydroxylation of eRF1. Mol. Cell, vol. 53, 2014, pp. 645–654. 10.1016/j.molcel.2013.12.028 PMC3991326 24486019

[50] R. Chowdhury, *et al*. Ribosomal oxygenases are structurally conserved from prokaryotes to humans. Nature, vol. 510, 2014, pp. 422–426. 10.1038/nature13263 PMC4066111 24814345

[51] Scotti J.S. (2014). Human oxygen sensing may have origins in prokaryotic elongation factor Tu prolyl-hydroxylation. Proc. Natl. Acad. Sci. USA.

